# Lipidomic changes of cerebral cortex in aldehyde dehydrogenase-2 knock-in heterozygote mice after chronic alcohol exposure

**DOI:** 10.3389/fnmol.2022.1053411

**Published:** 2023-01-19

**Authors:** Li Xiao, Jin Xiang, Xinyu Liu, Lin Yang, Ying Wei, Shiyong Fang, Jing Li, Yi Ye

**Affiliations:** ^1^Department of Forensic Toxicological Analysis, West China School of Basic Medical Sciences and Forensic Medicine, Sichuan University, Chengdu, Sichuan, China; ^2^Clinical Pharmacology Lab, Clinical Trial Center, West China Hospital, Sichuan University, Chengdu, Sichuan, China; ^3^West China School of Basic Medical Sciences and Forensic Medicine, Sichuan University, Chengdu, Sichuan, China; ^4^College of Pharmacy, North Sichuan Medical College, Nanchong, Sichuan, China; ^5^School of Forensic Medicine, Wannan Medical College, Wuhu, China; ^6^Department of Cardiothoracic Surgery, University Medical Center Regensburg, Regensburg, Bavaria, Germany

**Keywords:** ALDH2, lipidomics, chronic alcohol exposure, cerebral cortex, knock-in

## Abstract

**Introduction:**

Alcohol is the main legal drug in the world, and excessive consumption of alcohol seriously damages the morphological structure and function of various organs. The insufficiency of an essential enzyme in ethanol metabolism, aldehyde dehydrogenase-2 (*ALDH2*), will aggravate the alcohol-induced brain injury. The effect of *ALDH2* after chronic alcohol exposure on global lipid profiling of the brain remains unclear.

**Methods:**

In this study, ALDH2*2 knock-in mice were fed the Lieber-DeCarli liquid diet containing ethanol for 8 weeks. Blood alcohol and acetaldehyde levels were examined, and the mice were tested through novel object recognition and the Y-maze test to evaluate cognitive impairment toward the end of the study. The lipidome profiling of cerebral cortex samples was investigated using a lipidomics method based on ultra-high performance liquid tandem chromatography quadrupole time of flight mass spectrometry (UHPLC-QTOFMS).

**Results and Discussion:**

Compared with similarly treated wild-type (WT) mice, ALDH2*2 mice exhibited poor cognitive performance, though the result did not achieve statistical significance. The lipidomics results indicated that 74 differential lipid species were selected in WT mice, of which 57 species were up-regulated, and 17 were down-regulated. Moreover, 99 differential lipids were identified in ALDH2*2 mice, of which 73 were up-regulated, and 26 were down-regulated. For ALDH2*2 mice, the number of changed significantly glycerophospholipids (GPs) subtypes was lower than that of WT mice. Interestingly, compared with WT mice, a lower proportion of polyunsaturated fatty acids (PUFAs) was found in ALDH2*2 mice. Collectively, the results provide clear evidence for a lipidomic signature of marked changes in the cerebral cortex of ALDH2*2 mice after chronic alcohol exposure.

**Highlights:**

• The cerebral cortex of heterozygous ALDH2*2 mice showed more significant changes in lipidome profiles after chronic alcohol exposure than wild-type mice.

• Most lipids were significantly up-regulated in both groups of mice, whereas the increase in TAG was restricted to WT mice.

• For ALDH2*2 mice, GPs substances changed significantly, and SHexCer and SM subclasses in sphingolipids also deserved attention.

## 1. Introduction

Alcohol drinking results in significant health problems while affecting the morphological structure and function of various organs (Rehm et al., 2009). Among the brain regions, the hippocampus and cerebral cortex show high sensitivity to the effects of alcohol, and the damage to the above areas is significantly correlated with impaired motor control and cognitive function (Rehm et al., 2009). Although clinical research has suggested that most alcoholics develop brain damage and degenerative diseases, the specific molecular mechanisms remain unclear (Zahr et al., 2011). Aldehyde dehydrogenase-2 (ALDH2) has been found as an indispensable enzyme in ethanol metabolism. It is generally known that an ALDH2-inactivating mutation (called ALDH2*2) is the most common single-point mutation in humans (Kimura et al., 2019). Furthermore, epidemiological results have suggested a relationship between this inactivating mutation and the increased propensity for common human brain pathologies after chronic alcohol exposure (Chen et al., 2014; Kimura et al., 2019).

Lipids are vital components of biological membranes. Moreover, they play a role in the regulation of cellular metabolism and homeostasis as signaling molecules (Huang and Freter, 2015). Brain tissue is characterized by high lipids content, and it contains different lipid species and molecules (Albouery et al., 2019). It has been confirmed that lipids account for nearly half of the dry weight of the brain, and they primarily comprise phospholipids, cholesterol, as well as sphingolipids (Albouery et al., 2019). Lipids play a critical role in maintaining the structure and function of the brain because of their high diversity and hydrophobicity (Han, 2007). Several reports indicate that lipid modifications are likely to affect brain development and increase the risk of cognitive impairment, and lifelong neurodegenerative diseases (Burgess et al., 2000; Bennett and Rakheja, 2013; Naudi et al., 2015). Recent research has suggested that chronic alcohol exposure significantly changes lipid profiles in the serum, prefrontal cortex, as well as striatum of rats (Li et al., 2018; Wang et al., 2019). Alcohol damage to the brain has been extensively studied. One of the reasons for this damage is that the accumulation of adducts formed by toxic metabolites of alcohol in the body can disrupt lipid membrane structure and mitochondrial function (Cui et al., 2019). Interestingly, ALDH2 plays a role in the detoxification of the above adducts, and it has been demonstrated that individuals with ALDH2 deficiency have a higher sensitivity to Alzheimer’s disease (AD; Chen et al., 2019). Some groups have constructed cognitive impairment and AD models using Aldh2^−/−^ mice generated by gene-targeting knockout (D’Souza et al., 2015). However, the overall level of ALDH2 gene expression in ALDH2*2 knock-in (KI) mice is not modified, and its enzymatic, structural defects, and phenotype can be the characteristics of human ALDH2*2 carriers. Accordingly, ALDH2*2 mice are an ideal model for the research of human diseases associated with ALDH2 deficiency (Chen et al., 2014). Our previous study also observed the accumulation of acetaldehyde in ALDH2*2 mice after acute alcohol administration, which confirmed the insufficiency of ALDH2 activity (Li et al., 2022).

The role of ALDH2 in the effects of alcohol on brain lipid profiles, especially chronic alcohol exposure, has been rarely studied though numerous studies have investigated the molecular mechanisms of alcohol-related brain injury. This study aimed to explore the effect of ALDH2 on the brain after chronic alcohol exposure. The effects of chronic alcohol exposure on the lipidome of the cerebral cortex in ALDH2*2 mice were analyzed using mass spectrometry-based lipidomes. The data of this study indicated that after chronic alcohol exposure, the cerebral cortex of ALDH2*2 mice had more significant changes in the composition and content of lipid classes than in WT mice, and the above changes were accompanied by the modifications of fatty acid saturation and carbon chain length, which may contribute to a relationship between the diverse species of lipids and brain damage and guide subsequent research on the ALDH2 mutant population.

## 2. Materials and methods

### 2.1. Mice

The ALDH2*2 mice on a C57BL/6 N background were kindly provided by Cyagen (Guangzhou) Biosciences Inc. and were further backcrossed to a C57BL/6 N background for at least eight generations in our facility. The offspring mice were obtained by the cross-breeding of heterozygous ALDH2*2 mice. They were divided into cages by gender and randomly numbered by ear tagging. About 1-2 mm of mouse tail tissue was cut from each offspring mouse, and DNA was extracted by TIANamp Genomic DNA Kit (TIANGEN, Beijing), followed by PCR amplification and polymorphism sequencing. WT littermates of ALDH2*2 mice were chosen as the control groups. The mice were housed on a 12 h/12 h light/dark cycle. Eight- to 10-week-old male mice were fed the Lieber-DeCarli liquid diet containing 4% ethanol for 8 weeks, which is in the same treatment dose as some previous studies (Kwon et al., 2014; Golub et al., 2015; Hsu et al., 2020). All mice survived after chronic alcohol feeding. All experimental processes were performed in accordance with the National Institutes of Health Guide for the Care and Use of Laboratory Animals and gained approval from the Ethics Committee of Sichuan University.

### 2.2. Blood alcohol and acetaldehyde concentration

Blood ethanol and blood acetaldehyde concentrations were examined to clarify the clearance of alcohol and its metabolites in ALDH2*2 mice. There were at least 5 mice in the respective group, i.e., the WT + Alc (*n* = 7) and the ALDH2*2 + Alc (*n* = 5) groups. Based on anesthesia, approximately 100 μL of the blood sample was collected from the mouse orbital venous sinus and was placed in a heparin sodium anticoagulated EP tube. After 50 μL of blood was prepared by using the optimized PCA method, the ethanol and acetaldehyde concentration in whole blood was determined by Headspace Gas Chromatography coupled with flame ionization (Xiong et al., 2014; Li et al., 2022).

### 2.3. Animal behavior tests

After chronic alcohol administration for 8 weeks, more than five mice in the respective group, i.e., the WT Control (*n* = 7), the WT + Alc (*n* = 7), the ALDH2*2 Control (*n* = 5) and the ALDH2*2 + Alc (*n* = 5) groups, were used in the behavior tests. Behavioral tests were performed on the mice 1 week later in the following order: Y-maze test followed by novel object recognition (NOR), with a one-week interval. The data were recorded using VisuTrack Rodent Behavior Analysis System (Shanghai XinRuan Information Technology Co., Ltd., Shanghai, China) with a camera. The detailed operation of this study is like that of the existing study (Golub et al., 2015), which is briefly described as follows.

#### 2.3.1. Y-maze

The mice were placed in the center of an opaque apparatus with three equal-length arms (35 cm length × 5 cm width × 10 cm height) and explored freely for 10 min. The mice staying only in one arm were excluded from the test. The spontaneous change was scored when the mouse visited all three arms without going into the same arm twice in a row. The free change rate was calculated as the ratio of actual to possible changes (expressed as the overall number of arm entries minus two) multiplied by 100 (Golub et al., 2015). The apparatus was fully cleaned with 70% ethanol between trials.

#### 2.3.2. Novel object recognition

The apparatus for the open field test comprised an open box (40 cm × 40 cm × 40 cm) with black walls, which was evenly illuminated by overhead light. A two-day protocol consisting of a training stage and a test stage was employed. During the training stage (Day 1), the mouse was exposed to two objects that were the same and placed in the center of the open box for 10 min. At the test stage (Day 2), a novel object replaced one of the familiar objects from the former day, and the mouse was explored for 10 min. The following formula calculated the recognition index (RI) (Antunes and Biala, 2012).


$$RI=\frac{E_{NO}}{E_{NO}+E_{FO}}\times 100\%$$

E_NO_, time of exploring novel objects; E_FO_, time of exploring the familiar object.

After the respective trial, both the objects and box were fully washed with 70% ethanol.

### 2.4. Lipid sample preparation and lipidomic assay

#### 2.4.1. Animal sample preparation

At least five mice per group died from cervical dislocation after the behavioral tests were completed, i.e., the WT Control (*n* = 7), the WT + Alc (*n* = 7), the ALDH2*2 Control (*n* = 5) and the ALDH2*2 + Alc (*n* = 5) groups. Following rapid subsequent brain removal, the cerebral cortex was dissected in accordance with the Brain Atlas (Kressel et al., 2020), frozen immediately in liquid nitrogen, and then stored at −80°C.

#### 2.4.2. Extraction and determination of lipid metabolites

Metabolite profiling was performed with brain tissues (five replicates at least from the respective group) based on the previously published protocols (Dunn et al., 2011; Zhao et al., 2019) (Biotree, Shanghai). 25 mg of the sample was weighed and then placed into an EP tube with 200 μL water. 480 μL extract solution (MTBE: MeOH = 5: 1) was added sequentially. After 30 s vortex, the samples were homogenized at 35 Hz for 4 min and then sonicated for 5 min in the ice-water bath. The homogenization and sonication cycle were repeated 3 times. Subsequently, the samples were incubated at −40°C for 1 h and then centrifuged at 3000 rpm for 15 min at 4°C. 300 μL of supernatant was transferred to a fresh tube and then dried in a vacuum concentrator at 37°C. Next, the dried samples were reconstituted in 100 μL of 50% methanol in dichloromethane by sonication for 10 min in the ice-water bath. Afterward, the constitution was centrifuged at 13,000 rpm for 15 min at 4°C, and 75 μL of supernatant was transferred to a fresh glass vial for LC–MS/MS analysis. The quality control (QC) sample was produced by mixing an equal aliquot of the supernatants from all the samples. QC samples were inserted into the analysis queue to examine the system stability and data reliability in the whole experiment.

LC–MS/MS analyses were performed using a UHPLC system (1,290, Agilent Technologies), equipped with a Kinetex C18 column (2.1 × 100 mm, 1.7 μM, Phenomen). The mobile stage A consisted of 40% water, 60% acetonitrile, and 10 mmol/l ammonium formate. The mobile stage B consisted of 10% acetonitrile and 90% isopropanol, and it was added with 50 mL 10 mmol/L ammonium formate for every 1,000 mL mixed solvent. The analysis was conducted with the elution gradient as follows: 0 ~ 12.0 min, 40% ~ 100% B; 12.0 ~ 13.5 min, 100% B; 13.5 ~ 13.7 min, 100% ~ 40% B; 13.7 ~ 18.0 min, 40% B. The column temperature was 55°C. The auto-sampler temperature was 4°C, and the injection volume was 2 μL (pos) or 4 μL (neg).

The QE mass spectrometer was used for its ability to acquire MS/MS spectra on the data-dependent acquisition (DDA) mode in the control of the acquisition software (Xcalibur 4.0.27, Thermo). In the above mode, the acquisition software can continuously evaluate the full scan MS spectrum. The ESI source conditions were set, including sheath gas flow rate as 30 Arb, Aux gas flow rate as 10 Arb, capillary temperature 320°C (positive), 300°C (negative), full MS resolution as 70,000, MS/MS resolution as 17,500, collision energy as 15/30/45 in NCE mode, as well as spray Voltage as 5 kV (positive) or ~ 4.5 kV (negative).

The raw data files were converted into files in mzXML format using the “msconvert” program from ProteoWizard. Peak detection was first performed for the MS1 data. The CentWave algorithm in XCMS was used for peak detection with the MS/MS spectrum. Lipid identification was performed through a spectral match using LipidBlast library. A complete list of the lipidomic data is presented in Supplementary Table S1.

#### 2.4.3. Multivariate data analyses

The SIMCA-P 16.0.2 software (Umeta, Umea, Sweden) was used for the multivariate statistical analysis. Principal component analysis (PCA) and orthogonal partial least-squares-discriminant analysis (OPLS-DA) were performed after the Pareto scaling. Lipid molecules with the highest impact on the group clustering were identified in the variable importance (VIP)-plots (VIP ≥ 1). The unpaired Student’s t-test (*p* < 0.05) to the chemical shifts was also used to evaluate the significance of each metabolite. There were significant differences in the metabolites between two groups which showed both VIP ≥ 1 and *p* < 0.05. The identical and different changed lipids between WT and ALDH2*2 group after alcohol treatment were presented by using a Venn diagram. Subsequently, the changes of differential glycerophospholipids components and the top 20 species in accordance with their *p* values after chronic alcohol exposure were analyzed in depth. Moreover, the length and saturation of the fatty chain in the GPs were examined to evaluate the effect of chronic alcohol feeding more comprehensively on the lipid profiling of mice with different genotypes.

### 2.5. Statistically analysis

All the statistical evaluations of lipidomic data described in this study were conducted based on relative abundances. The experimental data are expressed as the mean ± SEM. Comparisons were drawn using unpaired two-tailed Student’s t tests or two-way repeated measures ANOVAs as appropriate. The differences between groups achieved statistical significance at *p* < 0.05.

## 3. Results

### 3.1. Generation of ALDH2*2 mice

The G → A point mutation in the ALDH2*2 mice was confirmed by DNA sequencing (Supplementary Figure S1A). Some studies reported decreased acetaldehyde clearance in ALDH2 deficient mice following chronic alcohol exposure (Kwon et al., 2014; Jamal et al., 2016). As expected, our findings were consistent with theirs, also suggesting that alcohol and acetaldehyde levels were much higher in ALDH2*2 mice than in WT mice (Supplementary Figures S1B,C).

### 3.2. Body weight and cognitive state in ALDH2*2 mice after chronic alcohol feeding

The mice were fed the Lieber-DeCarli liquid diet with or without alcohol for approximately 8 weeks, and their change in body weight was carefully monitored to examine the long-term effects of alcohol feeding on mice. ALDH2*2 mice had a significant weight loss after being fed chronic alcohol in Supplementary Figure S2A.

Y-maze and NOR tests were performed to examine cognitive behavior of ALDH2 deficient mice after chronic alcohol exposure. In Y-maze tests, free change rate and cognition index data are presented in Supplementary Figures S2B,C. Regardless of whether alcohol-fed or not, the cognitive index of ALDH2*2 mice tended to decrease compared with that of WT group, whereas there was no statistical significance.

### 3.3. Lipidomic metabolites change in the cerebral cortex of mice caused by chronic alcohol feeding

**Figure 1:**
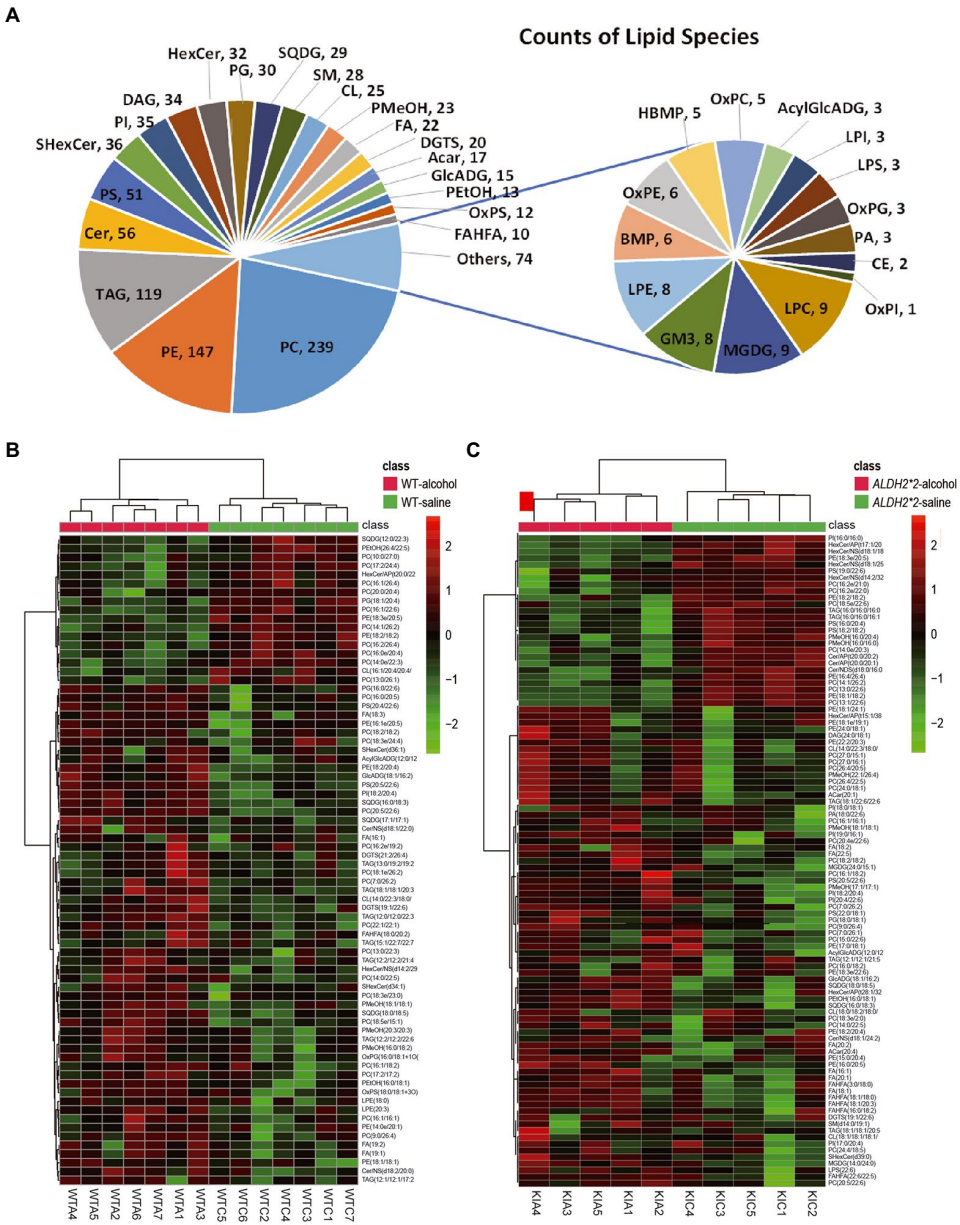
Alterations of the overall lipid composition in the cerebral cortex in response to chronic alcohol exposure. **(A)** Distribution of lipid classes used for subsequent analysis in all of the samples detected by LC–MS/MS. **(B)** Heatmaps of the significantly altered lipids (value of *p* < 0.05 and VIP > 1) in the cortex of WT mice (Saline vs. Alcohol-treated). **(C)** Heatmaps of the significantly altered lipids (value of *p* < 0.05 and VIP > 1) in the cortex of ALDH2*2 mice (Saline vs. Alcohol-treated). WTC and WTA represent saline and alcohol-treated cortex samples in wild-type mice, respectively. KIC and KIA represent saline and alcohol-treated cortex samples in ALDH2*2 mice, respectively.

The detected lipid subclasses and their categories are shown in Supplementary Figure S3A. More than 1,067 different lipid species were identified in the cortex, which comprising 239 phosphatidylcholines (PCs), 147 phosphatidylethanolamines (PEs), 119 TAGs, and other lipid classes (Figure 1A).

**Figure 2:**
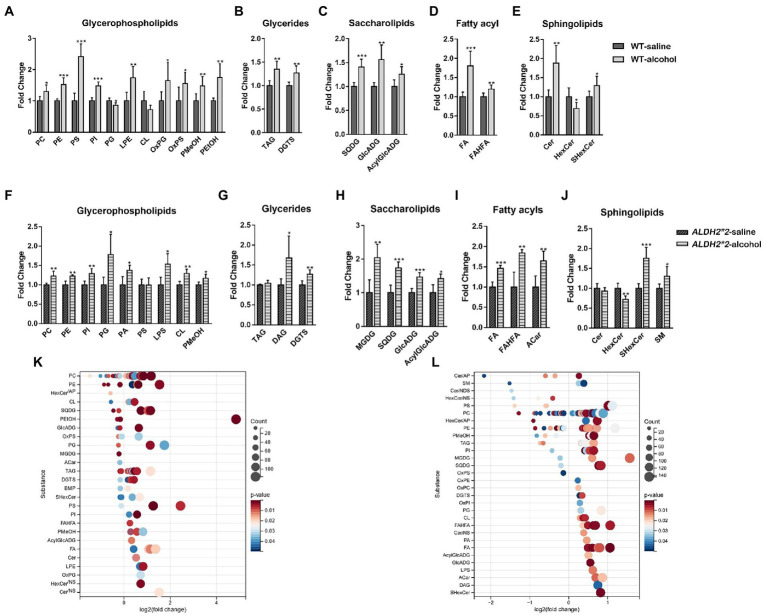
Alterations of the overall lipid distribution in the cerebral cortex in response to chronic alcohol exposure. **(A–E)** The intensity fold change of glycerophospholipids **(A)**, glycerides **(B)**, saccharolipids **(C)**, fatty acyls **(D)**, and sphingolipids in wild-type mice. **(F–J)** The intensity fold change of glycerophospholipids **(F)**, glycerides **(G)**, saccharolipids **(H)**, fatty acyls **(I)**, and sphingolipids **(J)** in ALDH2*2 mice. Data are presented as means + SEM (*n* = 5–7). *p < 0.05, **p < 0.01, and ***p < 0.001. **(K,L)** Log2 fold changes in lipid species in wild-type mice **(K)** and ALDH2*2 mice **(L)** after chronic alcohol exposure and the corresponding significance values displayed as value of *p*. Each dot represents a lipid species, and the dot size indicates significance. Only lipids with p < 0.05 are displayed (*n* = 5–7).

The principal component analysis (PCA) plots tended to be more segregated between saline and alcohol-treated groups in ALDH2*2 mice compared with WT mice (Supplementary Figures S3B,C). The orthogonal projections to latent structures discriminant analysis (OPLS-DA) plot showed a clear separation of two classes (Saline vs. alcohol-treated in WT group and ALDH2*2 group; Supplementary Figures S3D–F). From the OPLS-DA model, 74 features were selected that differentiated between the control and alcohol-fed in WT mice with variables important for the projection of VIP > 1.0 and *p* < 0.05, of which 57 were up-regulated and 17 were down-regulated (Figure 1B; Supplementary Table S2). With the same requirements, 99 differential lipid species were selected in the heterozygous ALDH2*2 mice, of which 73 were up-regulated and 26 were down-regulated (Figure 1C; Supplementary Table S2). Meanwhile, we also compared the alcohol-treated WT group and ALDH2*2 group. The results of the multivariate statistical analysis between the two groups were shown in Supplementary Figure S4. There were 26 up-regulated lipids and 99 down-regulated lipids in the ALDH2*2 group comparing to WT group after alcohol administration (Supplementary Table S2). Subsequently, we analyzed the segregation characteristics of the four groups and the differential lipids compared between the different groups (Supplementary Figure S5). The Venn diagram analysis disclosed that the number of uniquely altered differential lipids was 86 in the ALDH2*2-alcohol group, significantly more than the other three groups (Supplementary Figure S5C). Relative quantification results showed that chronic alcohol exposure significantly up-regulated the contents in WT mice as follows: the phosphatidylcholine (PC), phosphatidylethanolamine (PE), phosphatidylserine (PS), phosphatidylinositol (PI), lysphosphatidylethanolamine (LPE), oxidized Phosphatidylglycerol (OxPG), oxidized phosphatidylserine (OxPS), phosphatidyl methanol (PMeOH) and phosphatidyl ethanol (PetOH) in glycerophospholipids (Figure 2A); the triglycerides (TAG), diacylglycerol trimethyl homoserine (DGTS), sulfoquinovosyldiacylglycerol (SQDG), glucuronic acid diacylglycerol (GlcADG) and acyl glucuronic acid diacylglycerol (AcylGlcADG) in glycerides and saccharolipidis (Figures 2B,C); the fatty acid (FA) and branched fatty acid esters of hydroxy fatty acid (FAHFA) in fatty acyls (Figure 2D); the ceramides (Cer) and sulfatides hexosyl ceramide (ShexCer) in sphingolipids (Figure 2E).

The number of altered glycerophospholipid subclasses in ALDH2*2 mice was lower than that in WT mice. Although most glycerophospholipid subtypes showed consistent trends in both groups, subclasses such as PS, CL, PA, and LPS showed different changes in ALDH2*2 and WT mice (Figure 2F). Among glycerides, besides DGTS, DAG was also significantly up-regulated. In sphingolipids, Cer lipids tended to decrease, whereas SM significantly increased. Among saccharolipids and fatty acyl substances, except for the significant up-regulation of MGDG and Acar substances, the change trends of other substances were primarily the same as those of the WT mice (Figures 2G–J). To further probe individual lipid species that were regulated by chronic alcohol exposure, all the significantly changed lipid species were visualized using a bubble map. A total of 110 and 138 species were significantly changed in WT and heterozygous ALDH2*2 mice, respectively, using a value of p of 0.05 as a cutoff (Figures 2K,L). In general, the above findings suggested that lipid metabolism in the cortex of heterozygous mice after chronic alcohol-fed was more significantly affected.

### 3.4. The modifications of the composition and fatty acyl chain profiling of glycerophospholipids in the cerebral cortex of ALDH2*2 mice after chronic alcohol feeding

**Figure 3:**
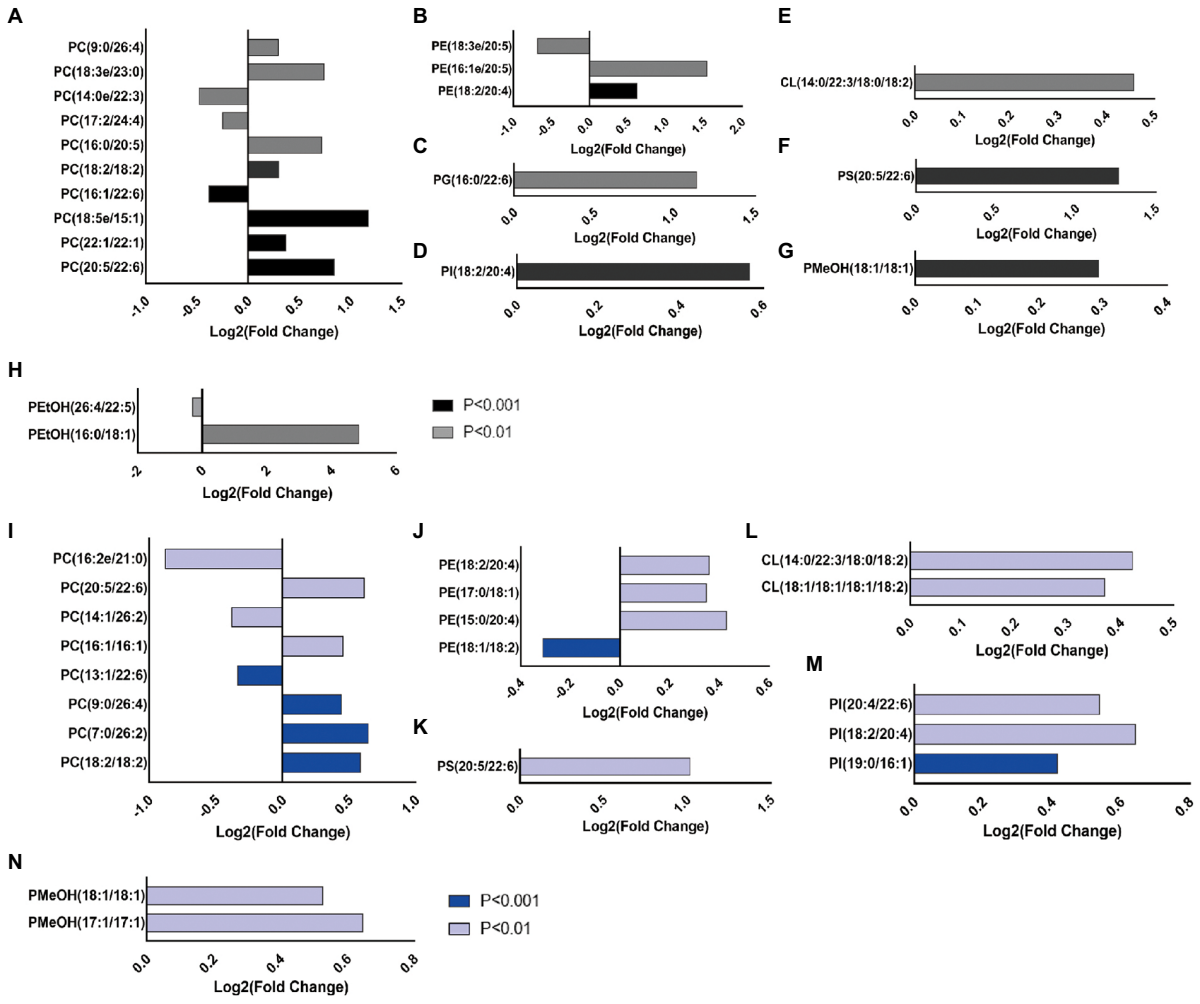
Chronic alcohol exposure alters the composition of glycerophospholipids and the top 20 GPs according to the value of *p* was showed (*n* = 5–7). **(A–H)** The intensity of individual fatty-acyl chains associated with different glycerophospholipid classes from wild-type mice. **(I–N)** The intensity of individual fatty-acyl chains associated with different glycerophospholipid classes from ALDH2*2 mice. The transparency of each bar is proportional to the significance values, which are displayed as *p* value. Light colors indicate *p* < 0.01, dark colors indicate *p* < 0.001.

Chronic alcohol administration enhanced the abundance of the different glycerophospholipid classes in WT and ALDH2*2 mice. Among the top 20 differential metabolites in glycerophospholipids, 15 lipid species significantly increased, and five lipid species had reserve changes in WT mice (Figures 3A–H). Sixteen glycerophospholipids were up-regulated, and four species were down-regulated in ALDH2*2 mice (Figures 3I–N).

**Figure 4:**
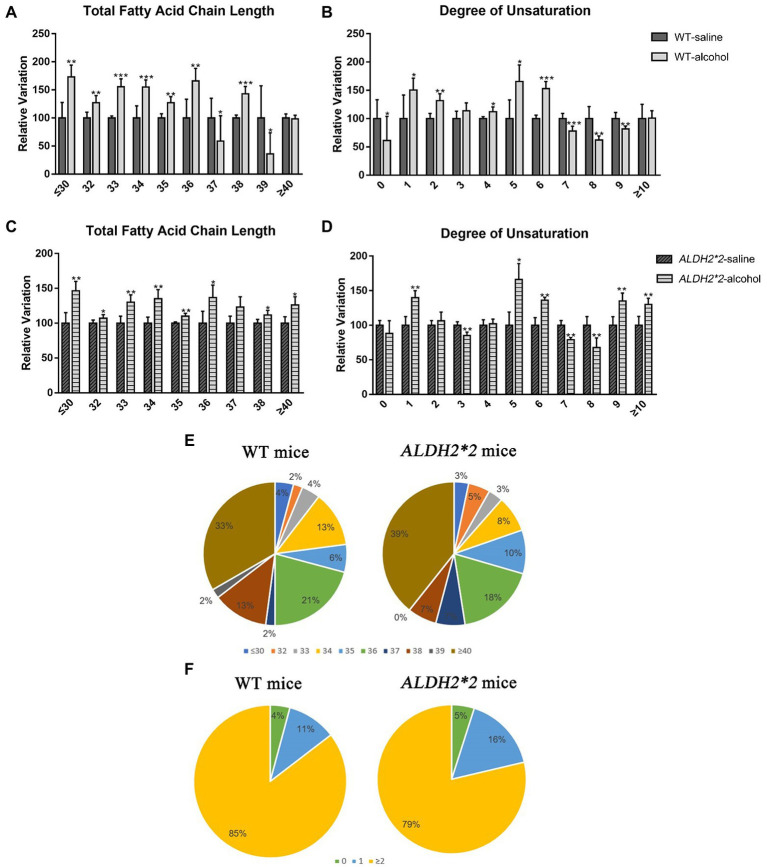
The modifications of lipid fatty acyl chain in GPs after chronic alcohol feeding. **(A,B)** Changes in fatty acid chain length and degree of unsaturation of GPs in cortex after chronic alcohol exposure in WT mice. **(C,D)** Changes in fatty acid chain length and degree of unsaturation of GPs in cortex after chronic alcohol exposure in ALDH2*2 mice. **(E)** Pie chart of proportion of different acyl chain lengths in wild-type and ALDH2*2 mice. **(F)** Pie chart of proportion of different carbon saturation in wild type and ALDH2*2 mice. *p < 0.05, **p < 0.01, and ***p < 0.001.

After chronic alcohol exposure, GPs species including most very long fatty-acyl chains significantly increased in WT and ALDH2*2 mice (Figures 4A–D). Fatty acyl groups with a carbon chain length greater than 40 accounted for the highest proportion in the two groups, and the proportion increased sequentially. However, the proportion of 36 carbon fatty-acyl chains had adverse changes (Figure 4E). Compared with WT group, ALDH2*2 mice had a significantly higher and lower percentage of saturated and polyunsaturated fatty acyls, respectively (Figure 4F). Furthermore, ALDH2*2 mice significantly increased in most unsaturated fatty acyl species (Figures 4D,F).

## 4. Discussion

In line with the studies on alcoholic fatty liver and atrial fibrillation studies (Kwon et al., 2014; Hsu et al., 2020), chronic alcohol-treated ALDH2*2 mice showed significant weight loss. The phenomenon is mostly likely to be an adverse reaction caused by the accumulation of acetaldehyde, which makes ALDH2*2 mice refuse to intake enough liquid diet. Existing research has confirmed that chronic ethanol intake causes long-term behavioral and memory impairments (Pascual et al., 2015). ALDH2, a vital enzyme in alcohol metabolism, is significantly related to alcohol dependence and degenerative diseases, whereas its specific neural molecular mechanism remains unclear. A previous study clarified the role of acetaldehyde in alcoholic brain injury by transgenic overexpression of ALDH2 in mitochondria, enabling a better understanding of mechanisms of alcoholic neuropathy (Ren et al., 2009). Unfortunately, there was no significant difference in cognitive behavior observed in ALDH2 deficiency mice after chronic alcohol administration in this study. Besides for ALDH2 genotype, the severity of deficits might be impacted by age, sex, schedule, and type of alcohol administration (Charlton and Perry, 2022). However, as the respect of lipid changes, ALDH2*2 mice had not only vital changes in the overall composition of lipids, but also diverse alterations in the length and degree of unsaturation of the acyl chains of glycerophospholipids. The most preferentially changed lipids were GPs, GLs, and SPs lipids in this study. The brain contains a lot of phospholipids (glycerophospholipids and sphingomyelins; O’Brien and Sampson, 1965; Söderberg et al., 1990). Besides regulating membrane fluidity, dynamics, and homeostasis as major components of cellular membranes, glycerophospholipids play many other roles such as being reservoirs of signaling messengers and antioxidant molecules (Farooqui et al., 2000; Hishikawa et al., 2014; Tracey et al., 2018). PE and PC are the two most represented classes of phospholipids in the brain, notably in cortical tissue (Sastry, 1985; Little et al., 2007). Although most studies showed a reduction of both PC and PE in the brains of AD patients, there were still some conflicting findings (Whiley et al., 2014; Proitsi et al., 2017). Our study indicated that both glycerophospholipids were significantly increased after alcohol exposure in WT and KI mice groups after alcohol feeding.

Consistent with previous findings (Wang et al., 2019), chronic alcohol exposure widely modifies several lipids. Interestingly, most lipids were significantly up-regulated in both groups of mice, whereas the increase in TAG was restricted to WT mice, suggesting that reduced or deficient ALDH2 enzymatic activity may predispose to inhibition or change of TAG synthesis. TGs are the most predominant glycerides. Both clinical trials and animal experiments have confirmed that there is a causal relationship between TGs and cognitive impairment (Rogers et al., 1980; Farr et al., 2008). The reduction of TGs would make the test individuals show better cognitive ability. Besides, another study found an association between low-chain and very-low-chain triglycerides (LCTGs/VLCTGs) and AD, but there were no differences in serum TG between controls and AD patients (Proitsi et al., 2017). The results in this study were the opposite, and the increased glycerides were predominantly polyunsaturated, similar to past alcohol-induced changes in hepatic lipid metabolism (Israelsen et al., 2021). In addition, our study also indicated that ALDH2 may not cause brain damage mainly by affecting TGs after chronic exposure.

Ceramides are more than critical contributions to cell membrane structure, but mediate different fundamental cellular processes (e.g., cell proliferation to arrest, differentiation, apoptosis, or recognition of specific cellular response; Morales et al., 2007). ShexCers are formed by further sulfation of HexCers based on glycosylation of Cers (Ding et al., 2021). In ALDH2*2 mice, ShexCer as a critical lipid substance in maintaining the myelin structure of neurons, regulating axon-glial signaling (Shroff et al., 2009), and oligodendrocyte development and survival, more significantly increased than with the WT group. Autopsy analysis of AD brains revealed altered brain lipid profiles and disturbances in ceramide metabolism, suggesting that maintaining ceramide homeostasis is a prerequisite for normal brain function (Jazvinšćak et al., 2015). Elevated ceramide levels were mainly seen in patients with mild and moderate symptoms, particularly ceramides Cer16, Cer18, Cer20, and Cer24 (Filippov et al., 2012). In this study, the significantly up-regulated sphingolipids in ALDH2*2 mice after chronic alcohol exposure were mainly ShexCer and SM, which were ShexCer (d39:0) and SM (d14:0/19:1), respectively. Therefore, in future studies, for individuals with ALDH2 mutations, we can pay more attention to ShexCer and SM, to find out the relationship between the two types of sphingolipids and neurodegenerative diseases.

Extensive studies have suggested that changes in fatty acid chain length and saturation can increase oxidative damage to lipids and proteins, thus affecting nervous system homeostasis (Angelova and Abramov, 2018). In general, fatty acids have been classified into saturated (SFA), monounsaturated (MUFA), and polyunsaturated (PUFA) fatty acids, according to chemical structure and carbon chain length (Yehuda et al., 2005). Interest in polyunsaturated fatty acids (PUFAs) has escalated over the past few years due to their diverse roles in promoting health and reducing disease risk. Among the most studied PUFAs contain α-linolenic acid (ALA; 18:3 ω-3), stearic acid (SDA; 18:4 ω-3), and eicosapentaenoic acid (EPA; 20:5 ω-3), docosapentaenoic acid (DPA; 22:5 ω-3) and docosahexaenoic acid (DHA; 22:6 ω-3) (Shahidi and Ambigaipalan, 2018). The effects of PUFAs on brain function have been confirmed elsewhere, which consist of modifications of membrane fluidity, the activity of membrane-bound enzymes, the number and affinity of receptors, the function of ion channels, the production and activity of neurotransmitters, as well as signal transduction (Yehuda et al., 2005; Shahidi and Ambigaipalan, 2018). Our study focused on the relevant conditions in GPs. The results found that significant changes in DHA (C22:6)-GPs were observed in both groups of alcohol-treated mice, whereas the direction of change was not consistent within the respective group. Typically, DHA levels decline progressively with age and AD patients also showed low DHA levels throughout the brain (Pottala et al., 2014). It is necessary to conduct further research to investigate which ALDH2 mediates alcohol-induced disturbances of lipidome profiling in the brain, such as targeted detection of several specific PUFAs to clarify the relationship between PUFAs and brain damage in different genotypes.

Moreover, this study suggested longer fatty acyl chains and higher unsaturation degrees in the two administration groups, consistent with previous findings (Wang et al., 2019). Interestingly, the proportion of SFAs increased sequentially from normal to ALDH2-deficient mice, while the opposite was true for PUFAs. SFAs may be related to the up-regulated expression of several apoptotic genes, pro-inflammatory markers, and decreased levels of brain-derived neurotrophic factor (BDNF) in the brain (Meireles et al., 2015, 2016). In general, high SFA significantly changes the permeability of the blood–brain barrier, which may lead to increased neuroinflammation through blood–brain leakage of peripheral proteins (Hargrave et al., 2016). Numerous studies have suggested that neuroinflammation takes on a critical significance to degenerative diseases (Takechi et al., 2013; Calsolaro and Edison, 2016). Therefore, the above results reveal that ALDH2 deficiency may modify the ratio between SFAs and PUFAs, affecting structures (e.g., the blood–brain barrier) and ultimately aggravating the occurrence of neuroinflammation.

## 5. Conclusion

Collectively, the chronic alcohol-induced changes of lipids in the cerebral cortex of ALDH2*2 mice at the level of the lipidome were determined using a non-targeted lipidomic approach. Although this study does not establish an animal model of significant cognitive changes, possibly due to inadequate treatment time or insufficient sample size, it still provides new insights into the possible mechanisms of alcohol-induced brain injury in ALDH2 deficiency mice and uncover some specific lipids alterations in the following. The major modified lipid subspecies comprise PC, PE, and TAG. ShexCer and SM in sphingolipids should attract certain attention. The length and saturation of fatty-acyl chains are associated with GPs. The availability of lipid profiles will provide more insights into the role of ALDH2 in brain injury, thus opening up opportunities for drug development and treatment of alcohol-induced neurological disorders. The multiple omics techniques are warranted to look for potential mechanism of brain injure in alcohol induced ALDH2 deficiency mice.

## Data availability statement

The original contributions presented in the study are included in the article/Supplementary material, further inquiries can be directed to the corresponding author.

## Ethics statement

All experimental processes were performed in accordance with the National Institutes of Health Guide for the Care and Use of Laboratory Animals and gained approval from the Ethics Committee of Sichuan University (K2021039).

## Author contributions

LX: conceptualization, data curation, methodology, validation, visualization, writing-original draft preparation, and writing-review and editing. JX: formal analysis, investigation, validation, writing—original draft preparation, and writing—review and editing. XL: formal analysis, validation, visualization, and writing—review and editing. LY: formal analysis, methodology, validation, and data curation. YW: investigation, validation, and data curation. SF: formal analysis, methodology, and data curation. JL: investigation, visualization, and data curation. YY: conceptualization, data curation, supervision, validation, project administration, funding acquisition, and writing—review and editing. All authors contributed to the article and approved the submitted version.

## Funding

This study was funded by the opening project of the Shanghai Key Laboratory of Forensic Medicine [grant number KF1909].

## Conflict of interest

The authors declare that the research was conducted without any commercial or financial relationships that could be construed as a potential conflict of interest.

## Publisher’s note

All claims expressed in this article are solely those of the authors and do not necessarily represent those of their affiliated organizations, or those of the publisher, the editors and the reviewers. Any product that may be evaluated in this article, or claim that may be made by its manufacturer, is not guaranteed or endorsed by the publisher.
