# Supplementary material for: Lipidomic changes of cerebral cortex in aldehyde dehydrogenase-2 knock-in heterozygote mice after chronic alcohol exposure
Source: Front Mol Neurosci. 2023 Jan 19;15:1053411. doi: 10.3389/fnmol.2022.1053411 (PMC9893510; doi:10.3389/fnmol.2022.1053411)
Supplement: Supplementary file 3 [file Data_Sheet_1.docx]

Fig S1.


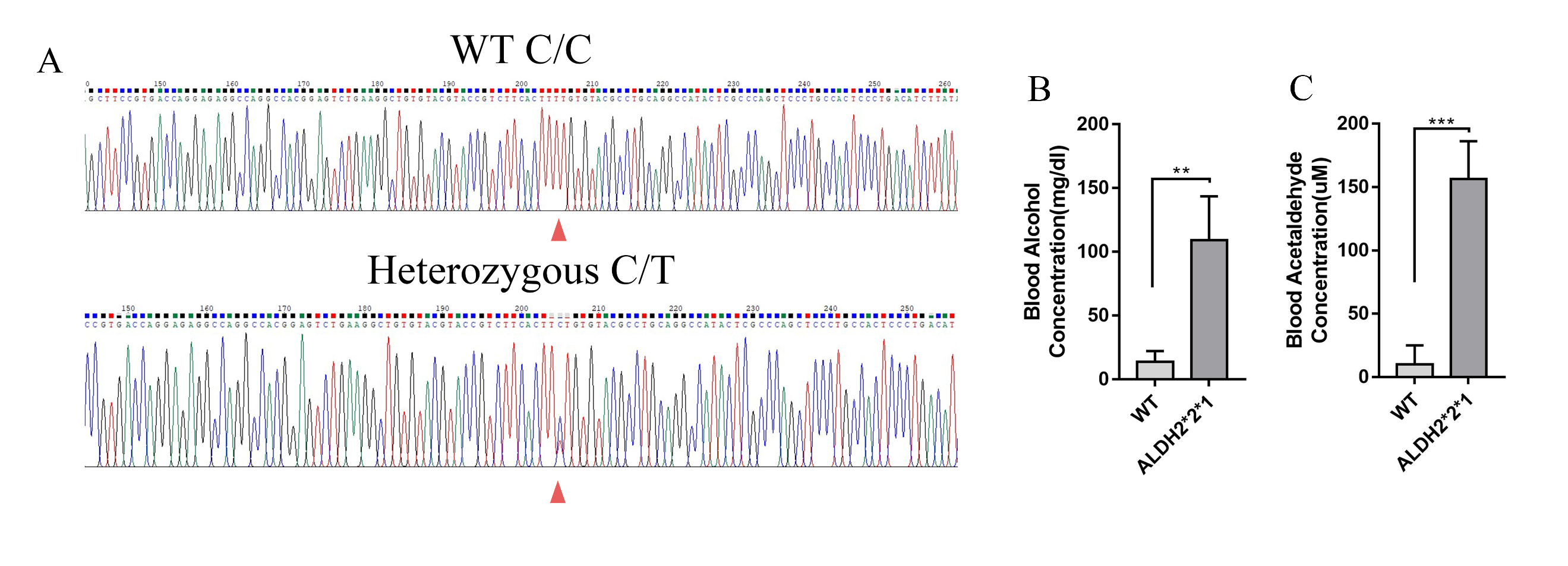


Fig S1. Genotyping and efficacy of Aldehyde dehydrogenase 2*2 knock-in (*ALDH2*2*) mice. (A) Mice containing the KI allele were confirmed by PCR amplification. C-to-T substitution in reverse sequence. (B) Blood alcohol concentrations. (C) Blood acetaldehyde levels. Error bars represent s.e.m. (n=7 in WT-treated group, n=5 in *ALDH2*2*-treated group) **p <0.01, ***p <0.001.

Fig S2.


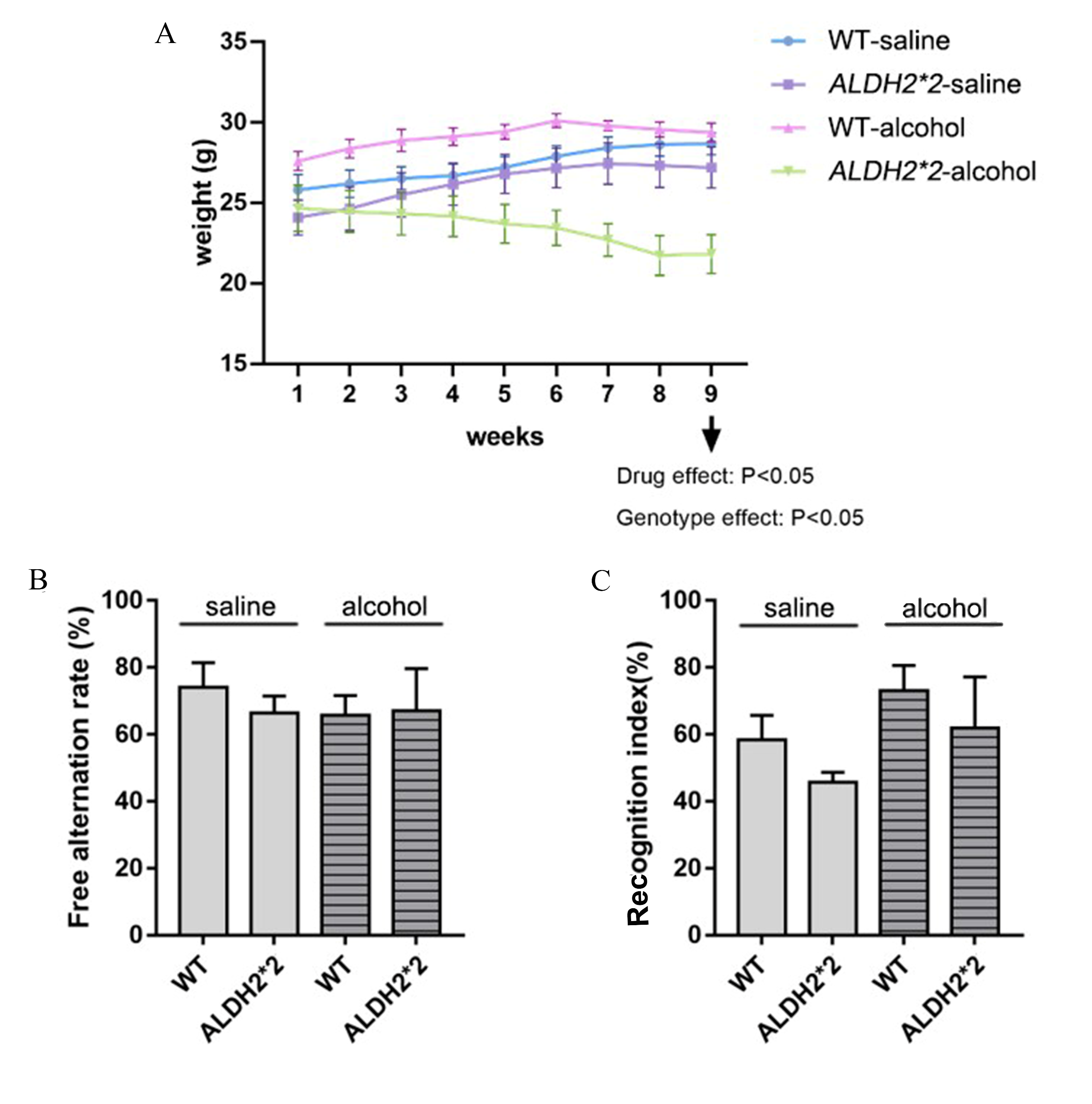


Figure S2. Chronic alcohol exposure for 8 weeks decreased the weight of *ALDH2*2* mice. (A) Chronic alcohol exposure for 8 weeks decreased the body weight in the *ALDH2*2* heterozygous mice (n= 5-7, two-way ANOVA: group × genotype interaction: F(1,10)=27.96, *p*=0.004). (B-C) Free alteration rate and recognition index of WT and *ALDH2*2* mice from saline and alcohol-treated (n=5-7). Error bars represent s.e.m.

Fig S3.


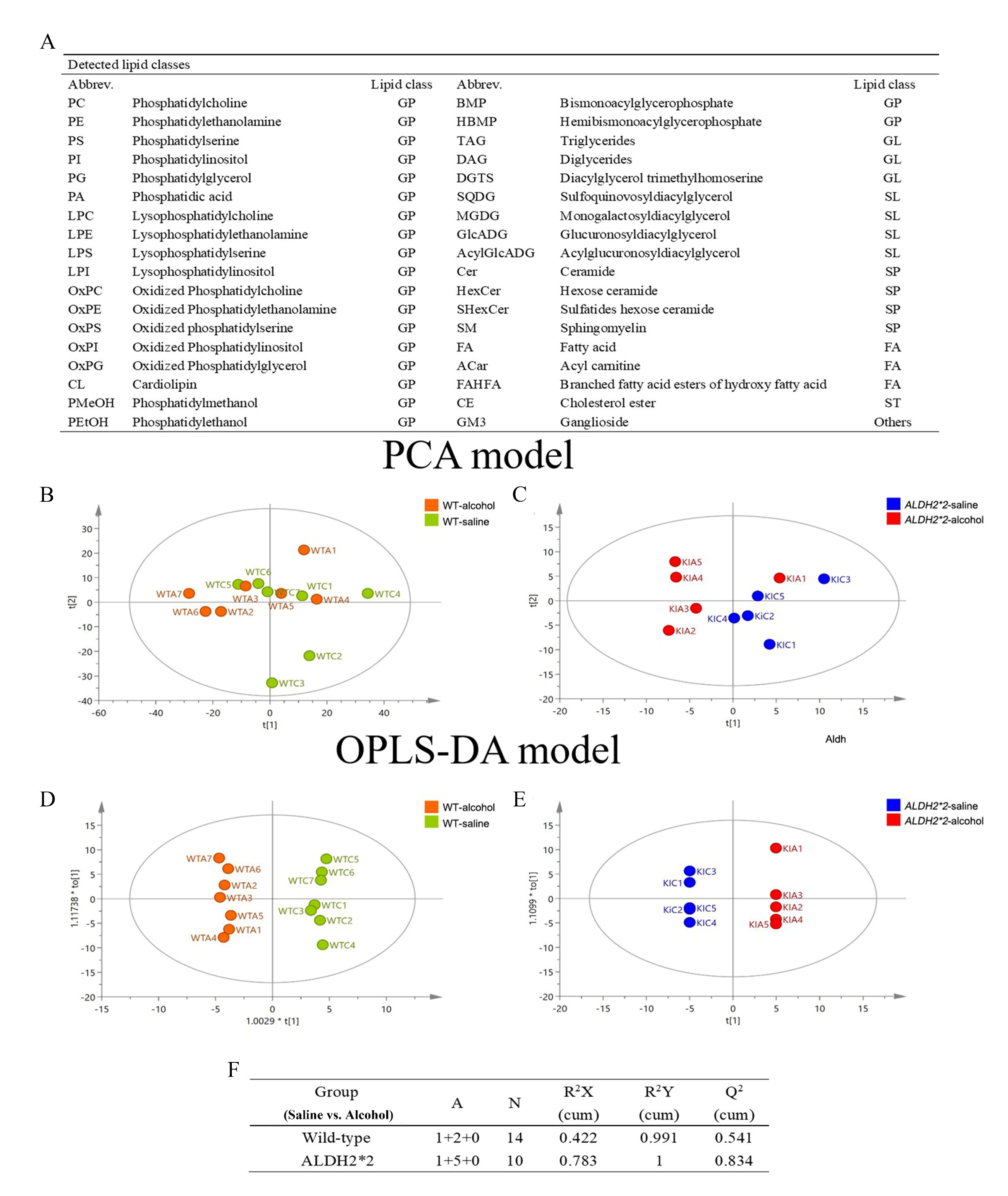


Figure S3. Multivariate statistical analysis for LC-MS lipidomic in saline and alcohol-treated cerebral cortex. (A) Detected lipid classes and their abbreviations of subclasses used throughout the paper (Note: GP, Glycerophospholipids; GL, Glycerides; SL, Saccharolipids; SP, Sphingolipids; FA, Fatty acyls; ST, Sterol lipids). (B-C) Unsupervised principal component analysis (PCA) scores plot. (D-E) Supervised orthogonal projections to latent structures discriminant analysis (OPLS-DA). (F) Validation of the OPLS-DA model.

Fig S4.


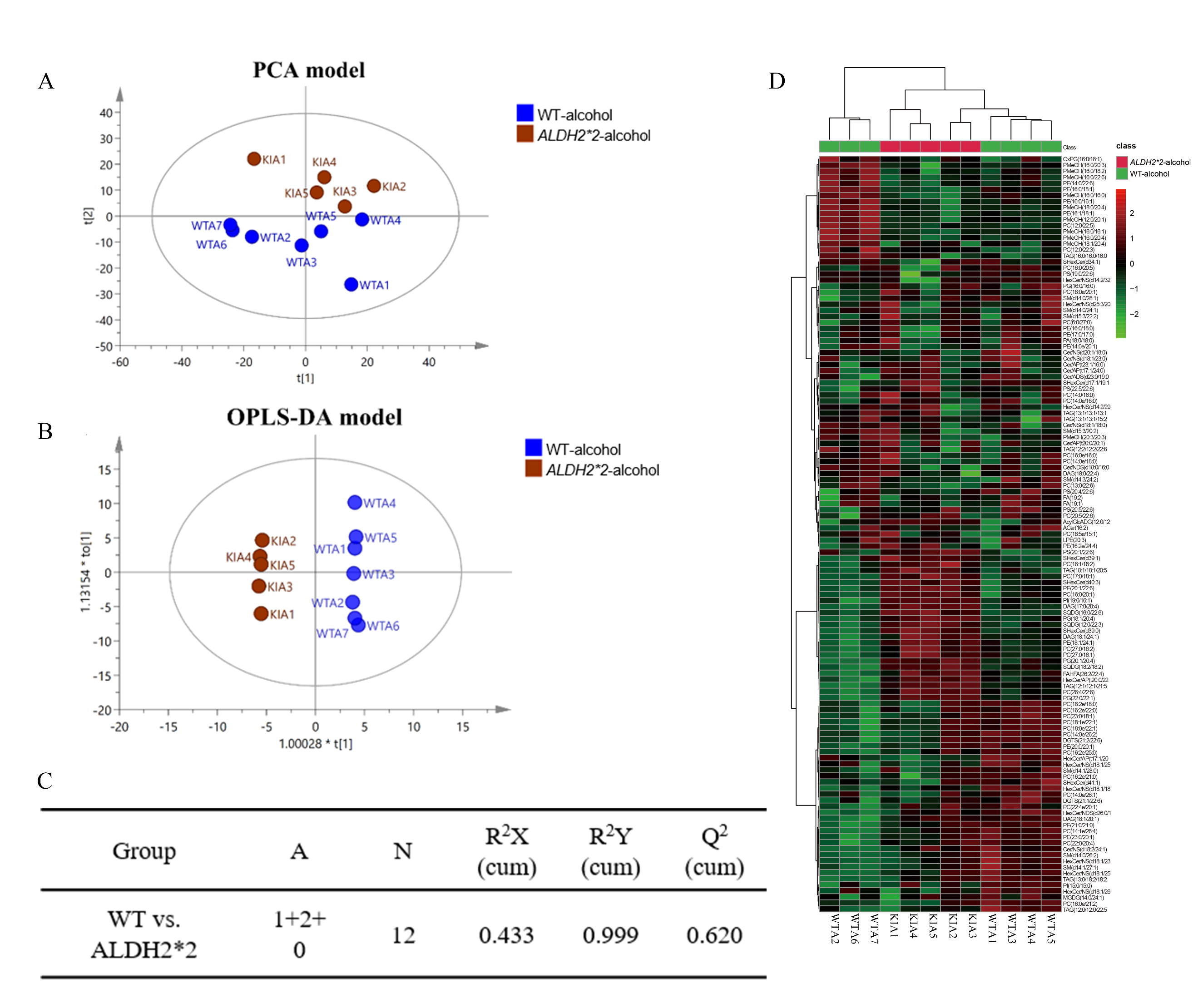


Figure S4. Multivariate statistical analysis for LC-MS lipidomic between wild-type and *ALDH2*2* mice after chronic alcohol exposure. (A) Unsupervised principal component analysis (PCA) scores plot. (B) Supervised orthogonal projections to latent structures discriminant analysis (OPLS-DA). (C) Validation of the OPLS-DA model. (D) Heatmaps of the significantly altered lipids (p-value < 0.05 and VIP > 1). WTA and KIA represent alcohol-treated cortex samples in wild-type mice and ALDH2*2 mice, respectively.

Fig S5.


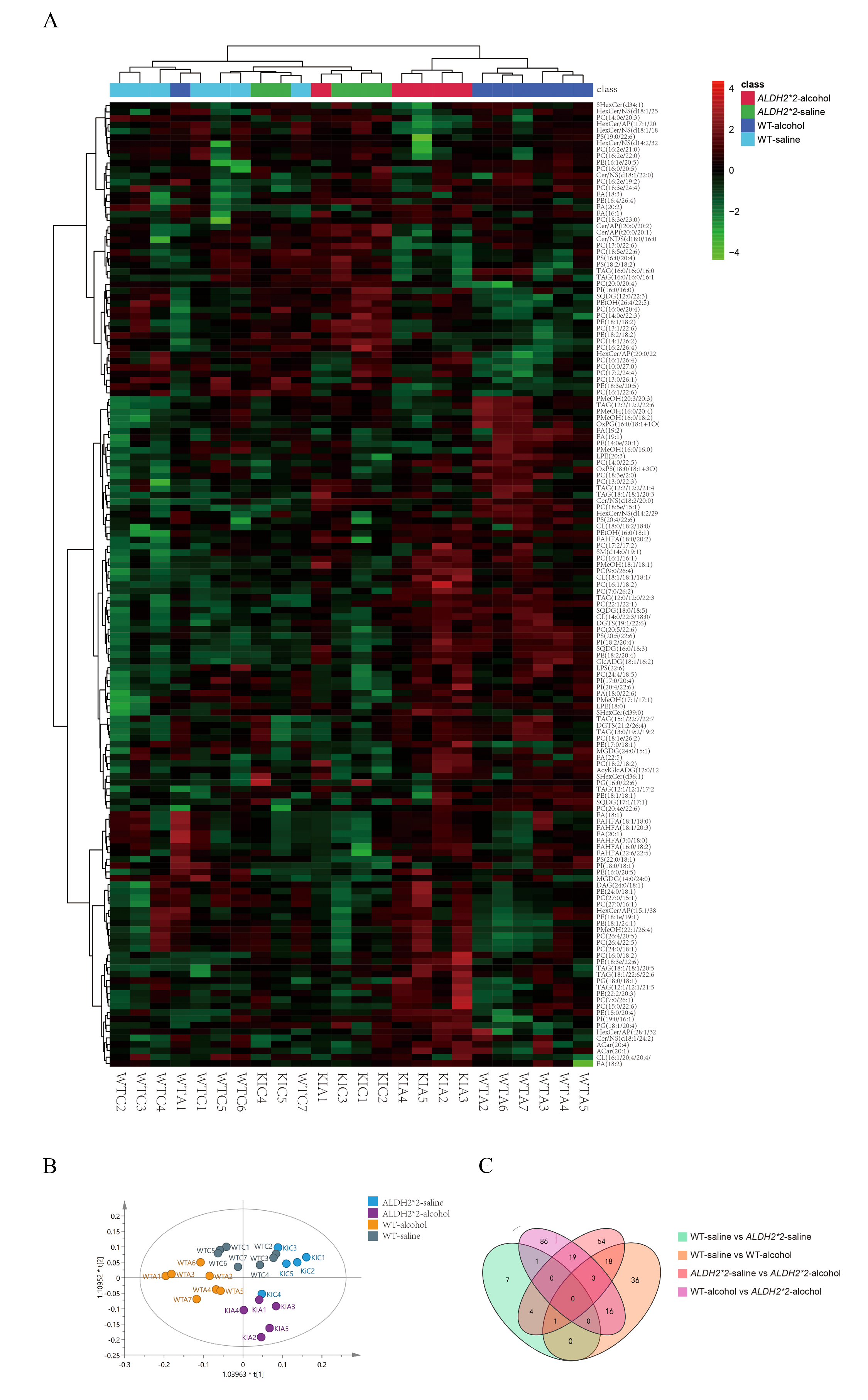


Figure S5. Characteristic analysis among the four groups. (A) Heatmaps of all significantly altered lipids after different comparisons. (B) Supervised orthogonal projections to latent structures discriminant analysis (OPLS-DA) among the four groups. (C) The Venn diagram of differential lipids after comparison between different two groups. WTC and KIC represent saline-treated cortex samples in wild-type mice and ALDH2*2 mice, respectively. WTA and KIA represent alcohol-treated cortex samples in wild-type mice and ALDH2*2 mice, respectively.
